# Comparative Transcriptome Profile between Iberian Pig Varieties Provides New Insights into Their Distinct Fat Deposition and Fatty Acids Content

**DOI:** 10.3390/ani11030627

**Published:** 2021-02-27

**Authors:** Ana Villaplana-Velasco, Jose Luis Noguera, Ramona Natacha Pena, Maria Ballester, Lourdes Muñoz, Elena González, Juan Florencio Tejeda, Noelia Ibáñez-Escriche

**Affiliations:** 1 Genetics and Genomics, The Roslin Institute, Royal (Dick) School of Veterinary Studies, The University of Edinburgh, Easter Bush Campus, Midlothian, Edinburgh EH25 9RG, UK; A.Villaplana-Velasco@sms.ed.ac.uk; 2 Centre for Medical Informatics, Usher Institute, The University of Edinburgh, 9 Little France Road, Edinburgh EH16 4UX, UK; 3 Animal Breeding and Genetics Program, IRTA, 25198 Lleida, Spain; joseluis.noguera@irta.cat; 4 Departament de Ciència Animal, Universitat de Lleida-Agrotecnio Center, 25198 Lleida, Spain; romi.pena@udl.cat; 5 Animal Breeding and Genetics Program, IRTA, Torre Marimon, 08140 Caldes de Montbui, Spain; maria.ballester@irta.cat; 6 INGA FOOD S.A, 06200 Almendralejo, Spain; l.munoz.v@nutreco.com; 7 Department of Animal Production and Food Science, Research University Institute of Agricultural Resources (INURA), Escuela de Ingenierías Agrarias, Universidad de Extremadura, 06007 Badajoz, Spain; malena@unex.es (E.G.); jftejeda@unex.es (J.F.T.); 8 Department for Animal Science and Tecnology, Universistat Politécnica de València, 46022 Valencia, Spain

**Keywords:** transcriptomics, Iberian pig, adipogenic potential, meat quality, RNA-seq

## Abstract

**Simple Summary:**

Iberian pigs are meat quality models due to their high fat content, high intramuscular fat, and oleic fatty acid composition. These parameters present great variability and are differentiated among the lines that make up the Iberian pig population. However, there is little information on how the genetic expression influences quality across Iberian varieties. This study aimed to compare the muscle expression profile between two varieties of Iberian pig (Torbiscal and Retinto) and their reciprocal crosses, differentiated by fatness. Our results suggest that the Retinto variety, which has the greatest fat content amongst the studied Iberian varieties, showed a higher expression of genes related to adiposity. Likewise, a higher expression of genes related to lipolysis was found in the Torbiscal variety, described as having less fat content than Retinto. Further genetic variation analysis in these Iberian varieties showed relevant associations for SNP (Single Nucleotide Polymorphism), related to these differentially expressed genes, with the meat quality traits. Thus, our findings evidence that differences in the genetic architecture and expression of Iberian varieties might explain the variability in their fat content and composition and hence, their meat quality.

**Abstract:**

The high deposition of intramuscular fat and the content of oleic fatty acid are characteristic of the Iberian pig. These two parameters present great variability and are differentiated amongst the varieties that make up the Iberian pig population. Although previous studies generated evidence for causal genes and polymorphisms associated to the adipogenic potential of the Iberian pig, there is little information about how genetic expression influences this trait’s variability. The aim of this study was to analyses the expression profile between two varieties of Iberian pig (Torbiscal and Retinto) and their reciprocal crosses differentiated in their intramuscular fat (IMF) content and fatty acid (FA) composition in the *Longissimus thoracis* muscle using an RNA-seq approach. Our results corroborate that the Retinto variety is the fattiest amongst all studied varieties as its upregulated genes, such as *FABP3* and *FABP5*, *SLC27A1* and *VEGFA* among others, contribute to increasing adiposity. In its turn, Torbiscal pigs showed an upregulation of genes associated with the inhibition of fat deposition such as *ADIPOQ* and *CPT1A*. Further genetic variation analysis in these Iberian varieties showed relevant associations for SNP located within the differentially expressed genes with IMF and FA content. Thus, the differences found in the genetic architecture and the muscle transcriptome of these Iberian varieties might explain the variability in their fat content and composition and hence, their meat quality.

## 1. Introduction

The Iberian pig has always been a meat quality model due to its high fat content and its fatty acids composition [1]. In fact, Iberian products are famous and appreciated for their flavor, which depends on the volatile components produced by fatty acid’s oxidation [2]. The quality of these products is influenced both by the genetic background of the Iberian breed and its nurture [1,2,3,4].

The Iberian pig population is structured in seven genetic varieties, which differ in their fatty acid profiles and intramuscular fat (IMF) content, as well as in their morphology and productivity [5,6]. Fat deposition is a heritable trait in Iberian pigs and these differences may be influenced by the genetic background of each variety [6]. For instance, the Torbiscal (TT) variety has a lower IMF content in the *Longissimus thoracis* muscle than Retinto (RR). Moreover, the Torbiscal variety has lower oleic and linoleic fatty acids content than Retinto [5,6]. Additionally, the reciprocal cross between both varieties (RT and TR) reveals a negative heterosis for IMF [6].

The genetic differences between Iberian pig varieties have been tackled by multiple approaches, some of which reported causal genes and polymorphisms contributing to pig breed differentiation [7,8]. However, there is scarce information on how gene expression influences the adiposity of Iberian pig varieties. Given the developments in next generation sequencing technologies, we sought to explore the muscle transcriptome differences between two Iberian pig varieties and their reciprocal crosses, which show distinct IMF content and fatty acid composition in *Longissimus thoracis* [6]. Using an RNA-seq approach, we identified those genes and metabolic pathways underlying the variation of their fat content and composition, and hence, their meat quality.

## 2. Materials and Methods

### 2.1. Ethics Approval and Phenotypic Records

#### 2.1.1. Ethics Approval

All animal procedures were carried out in accordance with the Spanish Guidelines for Animal Experimentation and were approved by the Ethical Committee of Institut de Recerca i Tecnologia Agroalimentàries (IRTA-2012-0054-C02-01).

#### 2.1.2. Phenotypic Records and Experimental Design

Twenty-eight castrated male Iberian pigs from the Retinto (RR) (*n* = 7) and Torbiscal (TT) (*n* = 7) varieties and their reciprocal crosses Retinto × Torbiscal (RT) (male × female) (*n* = 7) and Torbiscal × Retinto (TR) (*n* = 7) were used. The two varieties used in this study are recognized in Spain’s official Iberian herd book (Spanish Association of Iberian Purebred Pig Breeders, AECERIBER). During the experiment, pigs were kept from birth to slaughter under intensive rearing conditions such as those used in pig commercial farms, in the CAP-IRTA center (Pig Control and Evaluation) of Monells (Girona, Spain). All of them were reared in similar conditions to reach 102.8 ± 6.8 kg body weight (BW) at 242 ± 12.0 days of age. Then, each variety and cross were reared indoors ad libitum. After this fattening period, pigs were slaughtered in the commercial slaughterhouse “Jamón y Salud” of Llerena (Badajoz, Spain) at 299.3 ± 12.1 days of age and 153.5 ± 10.4 kg BW. For each animal, a sample of 200 g of *Longissimus thoracis* muscle was collected and stored at −32 °C until analyzed. The total IMF and the fatty acid profile were quantified according to the method described by [9].

### 2.2. RNA-Seq

#### 2.2.1. Sample Extraction and Sequencing Process

Twenty-eight tissue samples (seven per variety) were taken for RNA sequencing from the *Longissimus thoracis* muscle of the animals reared ad libitum. RNA was isolated by the acid phenol method using TRI-reagent (Sigma-Aldrich, Tres Cantos, Spain) and following the manufacturer’s instructions [10]. Then, the samples were pair-end sequenced using an Illumina Hiseq 2500 platform. On average, the number of reads was 11.79 ± 0.79 millions of reads per sample.

#### 2.2.2. Quality Control

The quality control of the reads was performed using FastQC v11.6 and MultiQC v1.5 [11,12,13]. Alignment and counting processes were carried out using STAR v2.4.0.1 package [14]. MultiQC software was used to visualize the reads before and after the quality control, and after aligning and counting the high-quality sequences.

#### 2.2.3. Differential Expression Analysis

The differentially expressed gene (DEG) analysis was performed with the R package edgeR [15]. Previously, genes whose counts were less than 10 reads were removed to prevent confounders in the normalization. Therefore, 16,252 protein-coding genes were analyzed in this study. Gene counts were normalized using the TMM (trimmed mean of M-values) method, which takes the sequencing depth and the total counts per sample. This procedure prevents sample collection and sequencing errors [14]. In the next step, for each gene, a negative binomial regression was fitted:

Y_ij_ = μ + L_i_ + b·P_ij_ + ε_ij_ (1)

where Y_ij_ is the normalized expression level of each gene, L_i_ is the variety of each sample, P_ij_ is the covariate, which corresponds to the BW at slaughter of each pig, and ε_ij_ is the residual term.

The statistical test used in this analysis was the empirical Bayes (EB), which enables contrasting the expression of each gene between varieties [14]. We implemented multiple test corrections using false discovery rate (FDR) [16]. Lastly, genes were considered differentially expressed (DEG) across groups when the fold change > 1.5 and the FDR < 0.1.

Gene ontology and metabolic pathway enrichment analyses were performed using David, GeneCards and the R package enrichR v1.0. [17,18]. Only those pathways with a *p*-value < 0.1 were considered.

### 2.3. Gene Variation Analysis and Association Study

In this analysis, SNP calling in coding and untranslated (UTR) regions of the top DEG (Table A3) was performed on the mapping files generated by STAR v2.4.0.1 package [14]. The BAM files were processed, and the variants were called using SAMtools v0.1.18 [19]. Only variants with a minimum root mean square (RMS) mapping quality of 30 were selected for further analysis. We also performed a visual inspection of alignments within the genomic regions of the genes most differentially expressed (Table A3) between varieties using the integrative genomics viewer (IGV) [20].

A selection of 12 SNP variants were genotyped in additional RR (44) and TT (74) pigs from an independent experiment [6,7]. The genotype was based on custom-design allelic discrimination assays (Applied Biosystems, Thermo Fisher Scientific, Waltham, MA, USA) in an OpenArray platform (Applied Biosystems). The association analysis between the SNPs and the IMF and their main fatty acid profiles were performed using plink 1.9 [21] and the statistical model included the sex as a fixed effect and age at slaughter as covariate and the two first principal components (PC) based on a previous PC analysis on this population with 50k SNPs [7].

## 3. Results and Discussion

### 3.1. RNA-Seq Analysis

#### 3.1.1. Quality Control

Quality control analysis revealed high-quality reads with a sequencing precision of 99%. The number of reads per sample after quality control did not vary significantly; at first, there were on average 11.74 ± 0.74 million reads and after the quality control it decreased to 11.65 ± 0.73 million. Moreover, the length of the reads was greater than 96 bp which does not differ from the sequencing read size (100 bp), and there was no adapter contamination higher that 0.1% at each sample. Lastly, the GC content of these samples (40–60%) was within the accepted CG content interval in Iberian pig [22].

The results of the alignment showed that the RNA sequences were correctly aligned with 94% of the sequence length mapped, whereas only 2% of the percentage of sequences aligned to multiple loci and short sequences and, for gene counts, 75% of genes were counted at each sample, and the number of genes mapped to multiple loci and unmapped was approximately 5%.

#### 3.1.2. Differential Gene Expression Analysis

From a total of 16,252 expressed genes, 3799 unique genes were differentially expressed (DEG) across the Iberian pig varieties and reciprocal crosses (RR, TT, RT, and TR) (Table 1). The RR variety had the greatest number of DEG compared to others (Table 1). In particular, the biggest divergences were found between RR and TR varieties followed by the contrast between RR and TT. Otherwise, the comparison of genetic expression levels between the TT, RT and TR resulted in a much lower number of DEG (Table 1). These results are in line with previous phenotypic analysis (6) which indicated a line, maternal/paternal and heterosis effects between these strains (RR and TT) for IMF and fatty acids composition. Additionally, despite of the small sample size (seven individuals) of our phenotypic data, a difference in IMF was found between RR and TR (−3.11, *p*-value 0.03) which could explain the greatest number of DEG found between these two crosses. This result would also suggest a possible paternal imprinting of the Torbiscal variety, but it cannot be validated with the current analysis and is falling outside the scope. Nevertheless, the DEG and phenotypic results showed that the RR variety is clearly distinct from the other variety and reciprocal crosses [6]. Thus, given the distinctive nature of the RR variety, our study has focused on analyzing the expression divergence between RR and the rest of Iberian genetic types analyzed in this study.

#### 3.1.3. Gene Ontology and Pathways Enrichment Analysis

Gene ontology and pathways enrichment analysis identified 16 metabolic pathways significantly associated with an adjusted *p*-value < 0.1, most of which are related to the lipid metabolism (Table A2). In Table 2, we show three metabolic pathways with the highest number of DEG whose function is associated with lipids absorption, fatty acids synthesis and catabolism, and adipocyte differentiation. The influence of these three metabolic pathways in pig IMF content was previously reported in other independent studies that compared the fatness and fatty acid composition of different pig populations [22,23,24]. Interestingly, 30 DEGs, from a total of 220 genes associated with these lipid metabolic pathways, promoted lipid absorption, synthesis, and fat cell differentiation. In fact, most of these 30 genes were upregulated in the RR variety, except for the *ADIPOQ*, *CPT1A* and *SREBF1* genes, with some exceptions (Table A2).

These 30 genes can be assembled in multiple gene groups, such as *ACAD, ACAA, CPT, COX* and *FABP*. The first four gene families are involved in the fatty acid beta-oxidation, either in the first reactions, carrying these molecules through the different membranes of the mitochondrion (*CPT*), or in the latest reactions (*ACAD*, *ACAA* and *COX*). Specifically, within these gene groups, we found that *ACADL*, *ACADS*, *ACADVL*, *ACAA1*, *ACAA2*, *COX1*, *COX2*, *COX3*, *COX4I1*, *COX4I2*, *COX5B*, *COX6A1*, *COX6A2*, *COX7A1*, *COX7A2*, *COX7B*, *COX7C*, *CPT1B* and *CPT2* genes were upregulated in the RR variety, when compared to the other genetic types analyzed, while *CPT1A* was downregulated. Knockout studies using Acadl^tm1Uab^ and Acadvl^tm1Vjeque^ mice showed that the lack of the coding proteins of *ACADL* and *ACADVL*, respectively, caused lipidosis, hypoglycemia and an elevated content of fatty acids in serum [25,26]. Interestingly, the *CPT* family includes the downregulated *CPT1A* gene, which encodes a carrier protein that controls the entrance of long fatty acids in the mitochondrion, promoting the beta-oxidation. Still, DE analysis showed that *CPT1B* and *CPT2* genes were upregulated in the RR variety. These genes have an analogous function in the mitochondrion although *CPT1B* is specifically located in the muscle. Previous studies suggested that the expression differences between analogous genes, *CPT1A* and *CPT1B*, had an impact in the glucose and fat metabolism [27,28]. Thus, these results could explain why RR variety had a higher content of IMF, PUFA and SFA as their gene expression profile promotes reduced fatty acid catabolism [6].

The *FABP* family is responsible for uptaking fatty acids through the cell membrane of different tissues. Previous studies showed that genes within this family (*FABP3*, *FABP4* and *FABP5*) play a key role in the fatness differences between pig lines [24]. Interestingly, *FABP3* and *FABP5* genes were upregulated in the skeletal muscle of RR animals. These genes have a high affinity towards long fatty acids, like the oleic acid (C18:1, n9), which is one of the biochemical characteristics of the RR pigs [6,23]. In addition, a mice knock-out model, Fabp5^tm1Hota^, showed that this gene regulates adipocyte differentiation and reduces triglyceride concentration in plasma [29]. This result is in line with published comparative studies in “Duroc × white pig varieties” and “Iberian × Landrace cross” which reported that the upregulation of *FABP* family, was associated with higher IMF and backfat content, respectively [22,24,30,31,32]. Lastly, multiple sequence variants within the *FABP3, FABP4* and *FABP5* genes and near genomic regions have been associated with pig fatness, specifically with increasing FA and IMF content [23,32,33]. Thus, the upregulation of *FABP3* and *FABP5* genes in RR variety supports its higher fat content within its meat products and endorses that these genes are good candidates for meat quality traits, such as IMF and backfat content.

In addition to these families, the DGE analyses identified additional genes playing a relevant role in lipid metabolism. *LPL*, *PLA2G7* and *SLC27A1* genes carry lipids through tissues, cells, or organelles. Our DEG analysis reports that these three genes are upregulated in RR as compared to TT, RT, and TR. *LPL* participates in lipid’s regulation, such as HDL biosynthesis, fat content distribution and as a lipid carrier between lipid and carbohydrate metabolisms [34]. *PLA2G7* promotes the transport of oleic fatty acid, which is responsible for the exceptional quality of the Iberian products. The different expression of *PLA2G7* may explain the meat quality variations amongst these Iberian pigs. Lastly, *SLC27A1* is a long-chain fatty acid membrane transporter that is active in many cell types, and it is highly expressed in pig’s *gluteus medius*, *Longissimus dorsi*, diaphragm and heart muscles [35]. Previous studies with knockout mice for *SLC27A1* gene, Slc27^a1tm1Jkk^, showed that its deficit is linked with a decrease in the intramuscular fat deposition [36]. In addition, this gene was found to be upregulated in an RNA-Seq experiment comparing Iberian pigs and other white pigs (i.e., Duroc) [23]. *SHDB* and *VEGFA* are upregulated genes in the RR variety associated with the beta-oxidation of fatty acids and with angiogenesis, migration, and cellular growth [37], respectively. Different studies using knockout mice for the *SHDB* gene reported that the lack of its expression activates the energy metabolism, producing obesity resistance and the development of thyroid lipomatosis [38]. *VEGFA* gene has been widely studied due to its influence in many cellular processes, such as nutrient diffusion and angiogenesis. Previous studies reported that its upregulation is associated with a higher fat content in pig varieties [22,31]. Thus, the high expression of *SLC27A1* and *VEGFA* in Retinto variety might cause a major absorption and accumulation of IMF and subcutaneous fat in those pigs, respectively, which coincides with the phenotypic characteristics of this variety [6].

Our differential expression analyses also showed other downregulated genes in RR variety, such as *ADIPOQ* and *SREBF1*. *ADIPOQ* encodes a specific hormone of the adipose tissue, which controls energy homeostasis, glucose, and lipid metabolism, and *SREBF1* is a transcription factor responsible for lipid’s homeostasis. Previous experiments reported that the expression of the *ADIPOQ* hormone controls multiple metabolic routes in different cell and tissue locations. As an example, the upregulation of *ADIPOQ* has been associated with insulin-sensitizing and anti-lipotoxic phenotypes in pigs [39,40]. A knockout mice study (Adipoq^tmPesch^) showed that the lipid’s metabolism, especially beta oxidation, is deactivated and there was a reduction in the insulin sensitivity [41]. In pigs, published studies suggest that the differential expression of *ADIPOQ* and its receptors in two pig lines with different IMF content, such as “Landrace × Meishan” [42], “low × high IMF Duroc pigs” [43,44] and “Duroc × Landrace × Yorkshire” vs. Laiwu [45], is associated with differences in its IMF deposition. Specifically, these studies show that the expression level of the *ADIPOQ* gene is lower in those pig varieties or crosses with a higher IMF content. Then, the downregulation of the *ADIPOQ* gene in RR might be associated with an increase in fat deposition, whereas its upregulation in TT, RT, and TR genotypes might lead to a lower IMF content and to upregulating metabolic pathways associated with FA oxidation [46], which agrees with the upregulation of *CPT1A* in these varieties. Hence, the characteristic expression level of the *ADIPOQ* gene in these two varieties agree with their adipogenic potential, as the cells in RR are increasing its fat content and TT cells are stimulating FA metabolism.

**Figure 1:**
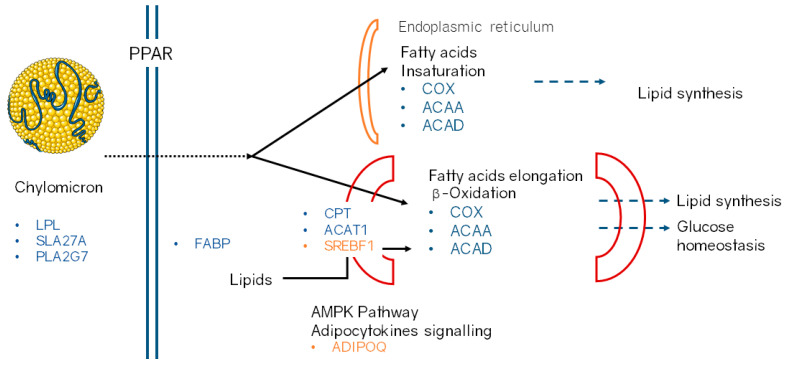
Differentially expressed genes clustered according to their associated metabolic pathways and cell location. We colored upregulated genes in Retinto variety in blue and downregulated genes in orange.

By clustering these DEGs according to their metabolic function and cellular location (Figure 1), we show how the muscle of RR pigs upregulates genes and metabolic pathways leading to an increased fat content. Specifically, RR upregulates genes involved in lipid transport and absorption from the plasma to the cell and to the organelles, like the endoplasmic reticulum and the mitochondrion, where the fatty acids are unsaturated and elongated, or oxidized.

### 3.2. Gene Variation Analysis and Association Study

Sequence analysis of the 30 DEGs identified 12 SNPs in UTR and coding regions (Table A3). These SNPs were genotyped in additional RR (44) and TT (73) pigs of an independent experiment with animals from the same populations (see [6] for more details) in which *Longissimus thoracis* IMF and their fatty acid profiles were also recorded. The association analysis showed that rs326546232 and rs346045742, SNPs located in *ADIPOQ* and *ACAT1* genes, respectively, had a significant association with meat quality traits (Table 3), particularly with saturated fatty acids like the stearic fatty acid, monosaturated fatty acids, like palmitoleic, and polyunsaturated fatty acids, like the linolenic acid. These SNPs were also associated with other fatty acids like oleic, the ratio between saturated and monosaturated fatty acids as well as the intramuscular fat.

Interestingly, RR pigs had distinctive allele frequencies in all the studied SNPs comparing with TT, (Table A3). The heterogeneity in these regions of the Iberian pig varieties genome might explain the diversity in the meat quality traits. Then, these results and its associations with the studied meat quality traits support our differential expression analysis previously described as it corroborates that the adipogenic potential of these Iberian genetic types is influenced by its genetic expression and architecture.

In summary, the profile of upregulated and downregulated genes in RR are related with an increasing fat absorption, deposit and metabolization in the muscle cells (Figure 1). In addition, SNPs located within multiple DEGs, which play a key role in the lipid’s metabolism, are more polymorphic in this variety and are also associated to the fatty acid profile of intramuscular fat, contributing to the nutritional value of meat. Thus, the genetic architecture and expression profile of this variety agrees with phenotypic studies that established that RR has the greatest adipogenic potential of the Iberian varieties [6].

## 4. Conclusions

The results of this experiment support that RNA-seq is a useful technique to elucidate which genes and pathways influence phenotypic differences between populations. In this study, we corroborate that Retinto pigs have the greatest fat content comparing to Torbiscal variety and their reciprocal crosses. This finding is supported by its gene expression profile as the upregulated and downregulated genes identified in this variety are associated with an increasing transport, absorption, differentiation, and accumulation of fatty acids in Retinto pigs. Thus, variation in gene expression profiles between these two varieties may explain the differences in their adipogenic potential. Remarkably, the different regulation of *ACAA*, *ACAD*, *CPT1B*, *LPL*, *FABP3*, *FABP5*, *SLC24A1*, *PLA2G7*, *SREBF1*, *SHDB*, *VEGFA* and *ADIPOQ* expression promotes the activation of anabolic routes in RR pigs, and the higher activation of beta-oxidation and catabolic routes in TT pigs.

Further gene variation and association analysis showed that the SNPs rs326546232 and rs346045742, located within *ADIPOQ* and *ACAT1* DE genes, respectively, are associated with all studied meat quality traits, like the saturation of fatty acids content which is related to the nutritional value of meat. In addition, these SNPs showed distinctive allele frequencies between RR and TT varieties, evidencing the polymorphic and then more diverse meat characteristics of Retinto.

Hence, variations in Iberian meat quality traits are influenced by the gene expression and genetic architecture of each breed variety. Although our study evidence that upregulated and downregulated genes and SNPs explain part of the meat quality traits variance between Iberian pig varieties, further studies are required to confirm that meat quality differences are mainly caused by these reported genes and genetic variants.

## Figures and Tables

**Table 1:** Number of differentially expressed genes between pig varieties.

* Variety/Number of Genes	RR vs. TT	RR vs. RT	RR vs. TR	RT vs. TR	TT vs. RT	TT vs. TR
Upregulated	883	374	1601	29	13	23
Not differentially expressed	14,685	15,499	12,690	16,203	16,200	16,208
Downregulated	684	379	1961	20	39	21

* RR: Retinto variety; TT: Torbiscal variety; RT: Retinto × Torbiscal crossing (male × female); TR: Torbiscal × Retinto crossing (male × female).

**Table 2:** Three most relevant metabolic pathways of the GO ^$^ enrichment analysis associated with lipid metabolism.

Metabolic Pathways	Gene Number *	P.Val Adj ^+^	Genes ^#^
**PPAR signaling pathway**	16/69	1.66 × 10^−2^	*SLC27A1*; *CPT1A*; *ADIPOQ*; *SCD5*; *ACSL5*; *APOA1*; *LPL*; *ACSL3*; *SORBS1*; *CPT1B*; *FABP3*; *CPT2*; *FABP5*; *ACADL*; *PPARA*; *ACAA1*
**Adipocytokine signaling pathway**	19/70	1.08 × 10^−6^	*CPT1A*; *CHUK*; *ADIPOQ*; *STAT3*; *ACSL5*; *PTPN11*; *IRS2*; *ACSL3*; *SLC2A4*; *TNF*; *CPT1B*; *PRKAG3*; *CAMKK1*; *TNFRSF1A*; *CAMKK2*; *MAPK9*; *AKT3*; *IKBKG*; *PPARA*
**Fatty acid degradation**	16/44	7.66 × 10^−6^	*GCDH*; *CPT1A*; *ACADVL*; *ECHS1*; *ACAA2*; *ECI2*; *ACSL5*; *ACSL3*; *CPT1B*; *ACAT1*; *ALDH3A2*; *HADHB*; *ACADL*; *CPT2*; *ACAA1*; *ACADS*

* Number of differentially expressed genes found in the pathway; ^+^ Bonferroni correction (P.Val adj); ^#^ Acronyms of the differentially expressed genes in each pathway; ^$^ Gene Ontology (GO).

**Table 3:** Genes interestingly associated (*p*-value < 2 × 10^−3^) with the main fatty acids of the intramuscular fat.

CHR ^€^	SNP ^£^	Bp ^$^	BETA *	STAT ^#^	*p*	Trait	Gene
9	rs326546232	36,542,668	0.3254	2.128	0.03549	Palmitol	*ACAT1*
12	rs318325536	38,825,225	−0.3934	−2.397	0.01821	Palmitol	*ACACA*
13	rs346045742	124,642,893	−0.3751	−1.993	0.04866	Palmitol	*ADIPOQ*
2	rs320952513	60,204,605	0.2027	2.147	0.03393	Palmitol	*SLC27A1*
9	rs326546232	36,542,668	−0.2323	−4.325	3.319 × 10^−5^	Palmitoleic	*ACAT1*
12	rs332506620	52,582,110	−0.1719	−2.839	0.005389	Palmitoleic	*ACADVL*
13	rs328315624	22,965,210	−0.1674	−2.493	0.01414	Palmitoleic	*ACAA1*
13	rs346045742	124,642,893	0.3222	4.929	2.916 × 10^−6^	Palmitoleic	*ADIPOQ*
2	rs343223441	4,272,429	0.3257	2.024	0.04538	Stearic	*CPT1A*
9	rs326546232	36,542,668	0.6782	5.136	1.198 × 10^−6^	Stearic	*ACAT1*
12	rs332506620	52,582,110	0.4897	3.228	0.001638	Stearic	*ACADVL*
13	rs346045742	124,642,893	−0.8342	−5.058	1.688 × 10^−6^	Stearic	*ADIPOQ*
9	rs326546232	36,542,668	−0.6153	−2.619	0.01005	Oleic	*ACAT1*
12	rs318325536	38,825,225	0.53	2.078	0.04004	Oleic	*ACACA*
13	rs346045742	124,642,893	0.6242	2.113	0.03687	Oleic	*ADIPOQ*
9	rs326546232	36,542,668	−0.2122	−2.585	0.01101	Linoleic	*ACAT1*
13	rs346045742	124,642,893	0.3031	3.015	0.003186	Linoleic	*ADIPOQ*
2	rs340138733	60,223,824	−0.02056	−2.25	0.03084	Linolenic	*SLC27A1*
9	rs326546232	36,542,668	−0.01461	−3.008	0.003244	Linolenic	*ACAT1*
12	rs332506620	52,582,110	−0.01111	−2.085	0.03938	Linolenic	*ACADVL*
13	rs328315624	22,965,210	−0.01629	−2.807	0.005892	Linolenic	*ACAA1*
13	rs346045742	124,642,893	0.02618	4.536	1.457 × 10^−5^	Linolenic	*ADIPOQ*
12	rs332506620	52,582,110	0.006203	2.042	0.04351	Arachidonic	*ACADVL*
9	rs326546232	36,542,668	1.012	3.717	0.0003164	SFA ^+^	*ACAT1*
12	rs332506620	52,582,110	0.6879	2.257	0.02597	SFA ^+^	*ACADVL*
13	rs346045742	124,642,893	−1.208	−3.567	0.0005336	SFA ^+^	*ADIPOQ*
9	rs326546232	36,542,668	−0.8293	−3.067	0.002714	MUFA ^+^	*ACAT1*
13	rs346045742	124,642,893	0.933	2.752	0.006916	MUFA ^+^	*ADIPOQ*
13	rs346045742	124,642,893	0.261	2.041	0.04358	PUFA ^+^	*ADIPOQ*
2	rs320952513	60,204,605	1.111	2.146	0.03406	IMF ^+^	*SLC27A1*
9	rs326546232	36,542,668	−0.9776	−3.21	0.00173	IMF ^+^	*ACAT1*
12	rs332506620	52,582,110	−0.7757	−2.314	0.02249	IMF ^+^	*ACADVL*
13	rs346045742	124,642,893	1.26	3.431	0.0008462	IMF ^+^	*ADIPOQ*
9	rs326546232	36,542,668	0.02975	3.413	0.0008961	SFA/MUFA ^+^	*ACAT1*
12	rs332506620	52,582,110	0.02001	2.061	0.04163	SFA/MUFA ^+^	*ACADVL*
13	rs346045742	124,642,893	−0.03416	−3.133	0.002212	SFA/MUFA ^+^	*ADIPOQ*

^+^ SFA: saturated fatty acids content; MUFA: monounsaturated fatty acids content; PUFA: polyunsaturated fatty acids contents; IMF: intramuscular fat content; SFA/MUFA: content of saturated fatty acids/content of monounsaturated fatty acids content; * BETA: SNPs effect associated with the studied trait in a linear regression; ^#^ STAT: value of the T-statistic; ^€^ CHR: Chromosome; ^£^ SNP: Single Nucleotide Polymorphism; ^$^ Bp: Base pair.

## Data Availability

The results from all data analysed during this study are included or displayed in this article (and its Table A1, Table A2 and Table A3, such as the Metabolic pathways, Fold changes of Differential expressed genes and Associated SNPs). However, other individual results may be made available from the corresponding author on request.

## Appendix A

**Table A1:** Metabolic pathways associated with lipid metabolism that include differentially expressed genes (DEG) of our study, adjusted *p*-value by Bonferroni correction (P.Val adj), number of DEGs in each pathway and DEG names.

Metabolic Pathways	P.Val Adj ^+^	DEG Number	Genes
Non-alcoholic fatty liver disease (NAFLD)	8.91 × 10^−65^	61/151	*COX7B*; *NDUFA13*; *NDUFA11*; *COX4I1*; *COX4I2*; *NDUFA10*; *PIK3CD*; *IRS2*; *FASLG*; *PIK3CB*; *COX6A1*; *TNF*; *COX6A2*; *COX7C*; *PRKAG3*; *CASP3*; *AKT3*; *IL6R*; *SREBF1*; *ADIPOQ*; *NDUFC2*; *NDUFC1*; *SDHD*; *SDHA*; *SDHB*; *TNFRSF1A*; *ERN1*; *NDUFS8*; *PIK3CA*; *NDUFS7*; *NDUFS5*; *DDIT3*; *UQCRC1*; *NDUFS3*; *NDUFS2*; *NDUFS1*; *PPARA*; *NDUFB8*; *NDUFB10*; *NDUFB11*; *NDUFB2*; *NDUFA4L2*; *COX7A2*; *UQCR10*; *COX5B*; *COX7A1*; *PIK3R5*; *MAPK9*; *BCL2L11*; *COX3*; *COX2*; *COX1*; *NDUFV1*; *NDUFA6*; *INSR*; *EIF2AK3*; *EIF2S1*; *IL6*; *ITCH*; *UQCRQ*; *CYTB*
PPAR signaling pathway	1.66 × 10^−2^	16/69	*SLC27A1*; *CPT1A*; *ADIPOQ*; *SCD5*; *ACSL5*; *APOA1*; *LPL*; *ACSL3*; *SORBS1*; *CPT1B*; *FABP3*; *CPT2*; *FABP5*; *ACADL*; *PPARA*; *ACAA1*
Glycerophospholipid metabolism	2.81 × 10^−2^	4/95	*PLA2G16*; *PLA2G4B*; *PLA2G10*; *PLD2*
AGE-RAGE signaling pathway in diabetic complications	6.97 × 10^−18^	29/101	*SERPINE1*; *PIK3CD*; *PIK3CB*; *TNF*; *FOXO1*; *PIK3R5*; *MAPK9*; *CCND1*; *CASP3*; *AKT3*; *PIM1*; *PLCG1*; *HRAS*; *EGR1*; *TGFB2*; *SMAD4*; *EDN1*; *NOS3*; *STAT3*; *PRKCA*; *MAPK12*; *VEGFA*; *MAPK11*; *IL6*; *PIK3CA*; *COL4A4*; *COL4A5*; *PLCD3*; *PLCD4*
Insulin signaling pathway	8.50 × 10^−24^	36/139	*PDE3B*; *CBLC*; *PIK3CD*; *CBLB*; *PPP1R3A*; *IRS2*; *SLC2A4*; *PIK3CB*; *CBL*; *FOXO1*; *PRKAG3*; *PIK3R5*; *GYS2*; *LIPE*; *MAPK9*; *PRKAR2A*; *AKT3*; *EIF4E*; *HRAS*; *SOCS4*; *SREBF1*; *PRKCI*; *EXOC7*; *INSR*; *BRAF*; *SORBS1*; *PHKA2*; *PPP1R3C*; *PRKAR1B*; *PIK3CA*; *PPP1R3E*; *HKDC1*; *RAPGEF1*; *CALM3*; *RAF1*; *SOS1*
Insulin resistance	1.96 × 10^−12^	26/109	*SLC27A1*; *PIK3CD*; *PPP1R3A*; *IRS2*; *SLC2A4*; *PIK3CB*; *TNF*; *FOXO1*; *PRKAG3*; *PIK3R5*; *GYS2*; *MAPK9*; *AKT3*; *SREBF1*; *CPT1A*; *NOS3*; *INSR*; *STAT3*; *PTPN11*; *CPT1B*; *TNFRSF1A*; *IL6*; *PPP1R3C*; *PIK3CA*; *PPP1R3E*; *PPARA*
Aldosterone-regulated sodium reabsorption	8.65 × 10^−4^	9/39	*PIK3CA*; *INSR*; *FXYD2*; *PIK3CD*; *PRKCA*; *ATP1A2*; *PIK3CB*; *ATP1A1*; *PIK3R5*
Linoleic acid metabolism	4.56 × 10^−4^	4/29	*PLA2G16*; *PLA2G4B*; *PLA2G10*; *ALOX15*
Adipocytokine signaling pathway	1.08 × 10^−6^	19/70	*CPT1A*; *CHUK*; *ADIPOQ*; *STAT3*; *ACSL5*; *PTPN11*; *IRS2*; *ACSL3*; *SLC2A4*; *TNF*; *CPT1B*; *PRKAG3*; *CAMKK1*; *TNFRSF1A*; *CAMKK2*; *MAPK9*; *AKT3*; *IKBKG*; *PPARA*
Arachidonic acid metabolism	1.11 × 10^−4^	6/62	*PLA2G16*; *GPX2*; *CYP2B6*; *PLA2G4B*; *ALOX15*; *PLA2G10*
Fatty acid degradation	7.66 × 10^−6^	16/44	*GCDH*; *CPT1A*; *ACADVL*; *ECHS1*; *ACAA2*; *ECI2*; *ACSL5*; *ACSL3*; *CPT1B*; *ACAT1*; *ALDH3A2*; *HADHB*; *ACADL*; *CPT2*; *ACAA1*; *ACADS*
Fat digestion and absorption	1.44 × 10^−2^	3/41	*SCARB1*; *PLA2G10*; *APOA1*
Sphingolipid metabolism	2.77 × 10^−3^	4/47	*CERS4*; *GAL3ST1*; *SPTLC1*; *SGMS1*
Phospholipase D signaling pathway	4.92 × 10^−6^	21/144	*INSR*; *PLA2G4B*; *ADCY4*; *ADCY3*; *PIK3CD*; *PRKCA*; *PTPN11*; *ADCY1*; *PIK3CB*; *ADCY8*; *ADCY6*; *PIK3R5*; *PLD2*; *PIK3CA*; *GRM7*; *AKT3*; *GNAS*; *PLCG1*; *RAF1*; *SOS1*; *HRAS*
Biosynthesis of unsaturated fatty acids	3.06 × 10^−3^	3/23	*SCD5*; *ACAA1*; *ACOT4*
Fatty acid elongation	2.61 × 10^−4^	4/25	*HADHB*; *ECHS1*; *ACAA2*; *ACOT4*

^+^ Bonferroni correction (P.Val adj).

**Table A2:** Fold change and FDR of differentially expressed genes relevant for the lipid’s metabolism in the two studied varieties and reciprocal crosses.

Gene	RR vs. TT ^+^		RR vs. RT		RR vs. TR		TT vs. RT		TT vs. TR		RT vs. TR	
	Fold Change *	FDR	Fold Change	FDR	Fold Change	FDR	Fold Change	FDR	Fold Change	FDR	Fold Change	FDR
*ACAA1*	2.17	0.009	2.198	0.012	2.554	3.940 × 10^−4^	1.013	0.99	1.177	0.78	1.162	0.83
*ACAA2*	1.891	0.004	1.525	0.068	1.6	0.013	0.806	0.593	0.846	0.678	1.05	0.936
*ACADL*	1.566	0.053	1.113	0.723	1.086	0.745	0.711	0.305	0.694	0.255	0.976	0.973
*ACADS*	1.832	0.005	1.457	0.105	1.576	0.019	0.795	0.551	0.86	0.71	1.081	0.886
*ACADVL*	1.713	0.001	1.216	0.266	1.356	0.028	0.709	0.096	0.791	0.323	1.116	0.741
*ACAT1*	1.548	0.07	1.2	0.514	1.518	0.041	0.775	0.515	0.981	0.98	1.265	0.601
*ADIPOQ*	0.414	0.068	0.58	0.3	0.51	0.11	1.4	0.715	1.233	0.828	0.881	0.915
*COX1*	1.869	0.002	1.323	0.215	1.798	0.001	0.707	0.233	0.962	0.947	1.36	0.346
*COX2*	1.991	4.498 × 10^−5^	1.356	0.151	1.88	2.041 × 10^−4^	0.681	0.156	0.944	0.905	1.386	0.268
*FABP3*	1.933	0.036	1.461	0.263	1.996	0.01	0.756	0.613	1.033	0.972	1.367	0.591
*FABP5*	1.507	0.083	1.018	0.962	1.251	0.283	0.676	0.202	0.83	0.637	1.229	0.633
*COX3*	1.951	0.001	1.509	0.047	1.979	6.130 × 10^−5^	0.773	0.421	1.014	0.983	1.312	0.407
*COX4I1*	2.067	3.681 × 10^−4^	1.665	0.018	2.202	1.46 × 10^−6^	0.805	0.546	1.065	0.898	1.323	0.423
*COX4I2*	2.561	1.882 × 10^−4^	1.874	0.015	1.99	0.001	0.732	0.46	0.777	0.561	1.062	0.929
*COX5B*	1.82	0.001	1.485	0.036	1.81	1.589 × 10^−4^	0.816	0.504	0.995	0.993	1.219	0.569
*COX6A1*	1.763	0.006	1.52	0.053	1.816	0.001	0.862	0.727	1.03	0.963	1.194	0.677
*COX6A2*	1.79	0.014	1.606	0.054	2.328	4.291 × 10^−5^	0.898	0.842	1.301	0.482	1.449	0.303
*COX7A1*	1.871	0.011	1.625	0.054	2.11	3.85 × 10^−1^	0.868	0.789	1.128	0.805	1.299	0.577
*COX7A2*	1.717	0.016	1.441	0.115	1.499	3.900 × 10^−2^	0.839	0.684	0.873	0.743	1.04	0.949
*COX7B*	1.809	0.003	1.477	0.06	1.939	1.03 × 10^−4^	0.817	0.558	1.072	0.879	1.313	0.405
*COX7C*	1.629	0.009	1.331	0.148	1.626	0.002	0.817	0.52	0.998	0.997	1.221	0.575
*CPT1A*	0.341	0.099	0.423	0.159	0.21	0.001	1.24	0.88	0.615	0.628	0.496	0.462
*CPT1B*	2.303	0.001	1.752	0.042	1.768	0.012	0.761	0.536	0.768	0.535	1.009	0.99
*CPT2*	2.134	0.002	1.331	0.305	1.431	0.117	0.624	0.167	0.67	0.27	1.074	0.911
*SDHB*	1.924	3.681 × 10^−4^	1.528	0.029	1.964	3.137 × 10^−5^	0.794	0.445	1.021	0.971	1.285	0.425
*LPL*	2.173	0.017	1.52	0.237	1.763	0.049	0.699	0.503	0.811	0.723	1.16	0.842
*PLA2G7*	1.94	2.985 × 10^−4^	1.999	9.831 × 10^−5^	1.59	0.003	1.03	0.956	0.82	0.496	0.795	0.471
*SLC27A1*	2.396	0.001	1.544	0.113	2.091	0.001	0.644	0.23	0.873	0.79	1.354	0.525
*VEGFA*	1.768	0.087	1.27	0.533	1.671	0.055	0.718	0.529	0.945	0.95	1.316	0.658
*SREBF1*	0.486	0.028	0.56	0.077	1.133	0.724	1.152	0.851	2.33	0.036	2.022	0.82

^+^ RR: Retinto × Retinto; TT: Torbiscal × Torbiscal; RT: Retinto × Torbiscal and TR: Torbiscal × Retinto; * Fold change: measure describing the expression difference between the contrasted varieties.

**Table A3:** Associated gene, identifier, location, reference allele, frequencies in Retinto (RR) and Torbiscal (TT) pigs of the 12 SNPs selected for genotyping.

Gene	SNP ^£^	Position	Chr ^€^	bp ^$^	REF AL *	freq RR ^#^	freq TT ^#^
*ACAA1*	rs328315624	Exon 16	13	22,965,210	C	0.22	0.56
*ACADVL*	rs332506620	3′UTR	12	52,582,110	T	0.22	0.63
*ADIPOQ*	rs346045742	Exon 2	13	124,642,893	A	0.59	0
*SLC27A1*	rs320952513	3′UTR	2	60,204,605	A	0.32	0
*SLC27A1*	rs340138733	Exon 3	2	60,223,824	T	0.21	0.55
*SLC27A1*	rs333601167	Exon 3	2	60,223,864	A	0.07	0.14
*CPT1A*	rs343223441	Exon 11	2	4,272,429	T	0.20	0.55
*CPT1A*	rs335135923	3′UTR	2	4,292,727	G	0.57	0.33
*CPT1B*	SSC5:155247	5′UTR	5	155,247	A	0.35	0.58
*LPL*	rs343665323	Exon 1	14	4,105,043	G	0.04	0.14
*ACAT1*	rs326546232	Exon 9	9	36,542,668	C	0.03	0.68
*CPT2*	rs345224133	Exon 4	6	159,041,404	C	0.40	0.56

^€^ Chr: Chromosome; ^£^ SNP: Single Nucleotide Polymorphism; ^$^ bp: Base pair; * REF AL: Allele of reference; ^#^ freq RR: frequency of the reference allele in RR variety; freq TT: frequency of the reference allele in TT variety.
